# Plasma-derived mitochondrial transplantation attenuates paraspinal muscle atrophy following spinal surgery

**DOI:** 10.1093/rb/rbaf090

**Published:** 2025-08-21

**Authors:** Ikhyun Lim, Seong-Hoon Kim, Mi Jin Kim, Chang-Koo Yun, Kyunghoon Min, Yong-Soo Choi

**Affiliations:** Department of Bioconvergence Science, Graduate School, CHA University, Seongnam 13488, Republic of Korea; Department of Rehabilitation Medicine, CHA Bundang Medical Center, CHA University School of Medicine, Seongnam 13496, Republic of Korea; Department of Bioconvergence Science, Graduate School, CHA University, Seongnam 13488, Republic of Korea; Department of Biotechnology, CHA University, Seongnam 13488, Republic of Korea; Department of Biotechnology, CHA University, Seongnam 13488, Republic of Korea; Department of Rehabilitation Medicine, CHA Bundang Medical Center, CHA University School of Medicine, Seongnam 13496, Republic of Korea; Department of Bioconvergence Science, Graduate School, CHA University, Seongnam 13488, Republic of Korea; Department of Biotechnology, CHA University, Seongnam 13488, Republic of Korea; Department of Life Science, Graduate School, CHA University, Seongnam 13488, Republic of Korea

**Keywords:** paraspinal muscle atrophy, muscle regeneration, laminectomy, plasma-derived mitochondria, mitochondrial transplantation

## Abstract

Paraspinal muscle atrophy (PMA) is a common complication after spinal surgery, often leading to reduced spinal stability and prolonged discomfort. While mitochondrial dysfunction has emerged as a key contributor to PMA, existing therapies do not adequately address this underlying pathophysiology. In this study, we investigated the regenerative potential of plasma-derived mitochondria (pMT) as a cell-free and autologous biomaterial to mitigate PMA. Mitochondria were isolated from human peripheral blood and confirmed to maintain their structural integrity and respiratory activity. In an *in vitro* model of muscle atrophy, pMT treatment improved cell viability, enhanced ATP production and restored mitochondrial function. In a rat model of surgery-induced PMA, intramuscular injections of pMT led to improved muscle morphology, including increased fiber cross-sectional area, along with reduced mechanical hypersensitivity. Transcriptomic analyses revealed that pMT transplantation modulated key pathways related to mitochondrial biogenesis and oxidative phosphorylation, while downregulating pro-apoptotic signals. These findings were corroborated by protein-level assessments showing restoration of muscle-specific markers and normalization of mitochondrial homeostasis. Taken together, this study highlights the therapeutic potential of pMT transplantation in addressing mitochondrial dysfunction and promoting muscle regeneration following spinal surgery. These findings suggest that pMT may serve as a minimally invasive, scalable and autologous regenerative approach to restore skeletal muscle integrity in clinically relevant contexts.

## Graphical abstract

**Figure:**
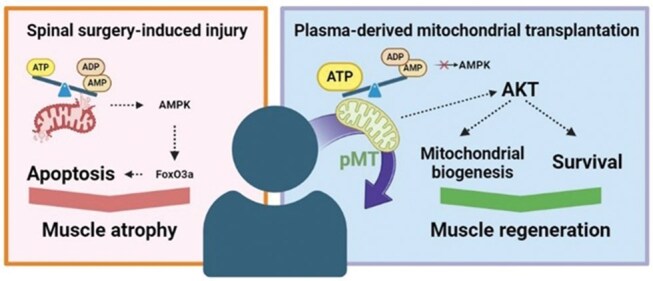

## Introduction

Paraspinal muscle atrophy (PMA) is a common complication after spinal surgery, often compromising spinal stability and impeding functional recovery [1–3]. Its clinical significance is further underscored by the association between persistent PMA and chronic postoperative pain and disability. Although a range of interventions—ranging from physical rehabilitation to biologic approaches—has been attempted, the effective restoration of paraspinal muscle structure and function remains challenging [2]. Current therapies, including cell-based treatments and platelet-rich plasma, offer partial benefits, but incomplete recovery is frequently observed, indicating the need for more effective regenerative strategies [4–6].

Mitochondria play a central role in skeletal muscle homeostasis, contributing to energy metabolism, proteostasis and redox balance [7, 8]. These functions are essential for muscle repair processes such as inflammation resolution, protein synthesis and tissue remodeling [9, 10]. As such, the regenerative capacity of skeletal muscle is closely linked to mitochondrial quality and dynamic remodeling. Following spinal surgery, mitochondrial dysfunction frequently occurs in paraspinal muscles, leading to impaired regenerative signaling, elevated oxidative stress and disrupted protein homeostasis—all of which contribute to the progression of PMA [11, 12]. Furthermore, the diminished mitochondrial function exacerbates muscle atrophy, highlighting the importance of restoring mitochondrial health as a therapeutic target [9].

In our previous studies, we showed that mitochondrial transplantation using mitochondria derived from cultured cells can attenuate muscle atrophy by enhancing mitochondrial energy metabolism and promoting regenerative signaling [13]. This approach delivers functional organelles directly to damaged tissue, bypassing upstream metabolic bottlenecks in dysfunctional cells. Subsequent research has strengthened the therapeutic rationale for mitochondrial transplantation in skeletal muscle recovery, demonstrating its capacity to restore ATP production and modulate stress responses [14–17]. Moreover, this approach complements existing treatments such as exercise, which exert beneficial effects primarily by stimulating mitochondrial biogenesis [14, 18, 19]. Nevertheless, the reliance on invasive tissue collection or prolonged cell expansion presents challenges for widespread clinical adoption.

Recent discoveries have identified the presence of cell-free mitochondria in human plasma, challenging earlier assumptions regarding their functional irrelevance [20–22]. In this study, we report that plasma-derived mitochondria (pMT) possess active respiratory enzyme function and structural integrity, rendering them a viable alternative to culture-derived mitochondria. In our preliminary investigation, we confirmed that pMT could be consistently isolated from both healthy individuals and patients with a range of chronic conditions, with no significant differences in concentration or respiratory function. Unlike conventional mitochondrial sources requiring cell culture or tissue biopsy, pMT can be obtained through simple blood draws, allowing for broader clinical scalability. Although further validation is warranted regarding the long-term behavior of pMT *in vivo*, their consistent detectability and function across individuals support their translational potential. As a naturally occurring, cell-free organelle population, pMT may overcome key translational barriers associated with cell-based therapies, including immunogenicity, manufacturing complexity and delivery logistics [23].

The primary objective of this study is to evaluate the efficacy of pMT transplantation in alleviating paraspinal muscle atrophy. Specifically, we aim to determine whether pMT can restore mitochondrial function, enhance muscle regeneration and improve structural outcomes in a rat model of PMA. This approach also enables a novel framework to investigate the therapeutic value of cell-free organelles in tissue repair. By elucidating the regenerative effects of pMT, this study may provide the foundation for future clinical strategies targeting mitochondrial dysfunction in broader musculoskeletal conditions, including sarcopenia and muscular dystrophy.

## Materials and methods

### Isolation of pMT

Peripheral blood was collected from healthy adult volunteers using EDTA-coated tubes, following approval from the institutional review board (IRB No. 2021-06-044). Plasma was obtained by centrifugation at 2000 rpm for 20 min at room temperature (∼22°C). The supernatant was subsequently centrifuged at 2000 × *g* for 10 min to remove residual platelets and cellular debris. To isolate mitochondria, the clarified plasma was then centrifuged at 20 000 × *g* for 10 min. All centrifugation steps were performed at room temperature to avoid cold-induced aggregation and maximize mitochondrial yield. The resulting mitochondrial pellet was resuspended in SHE buffer and stored at 4°C. For *in vitro* experiments, pMT preparations were applied immediately after isolation to maximize viability. For *in vivo* experiments, pMT were maintained at 4°C and administered within 1 hr of isolation. Additionally, long-term storage stability of pMT up to 6 months at 4°C has been validated using a patented storage formulation developed in our laboratory (Korean Patent Registered; PCT filed).

### Characterization of pMT

The structural integrity of pMT was examined by transmission electron microscopy (TEM). Samples were fixed in 2.5% glutaraldehyde with 0.1 M phosphate buffer (pH 7.3) at 4°C and post-fixed with 1% osmium tetroxide on ice for 2 hr. After dehydration and embedding in Epon 812, ultrathin sections (70 nm) were prepared using an UltraCut UCT ultramicrotome (Leica, Austria), stained with 2% uranyl acetate and lead citrate and visualized with a Tecnai G2 Spirit TWIN transmission electron microscope (FEI, USA) at 120 kV.

The size distribution of isolated pMT was analyzed using a particle analyzer (ELSZ-2000ZS; Otsuka Electronics, Japan), and mitochondrial content was quantified using a BCA assay (Pierce; Thermo Scientific, USA). To confirm mitochondrial identity and membrane potential, dual labeling with MitoTracker Green (Invitrogen, Waltham, MA, USA) and MitoTracker Red CMXRos (Invitrogen) was performed, followed by flow cytometric analysis. Mitochondrial purity was further assessed by Western blotting, targeting mitochondrial markers (e.g. COX IV, TOM20) and excluding non-mitochondrial components (e.g. p84, LAMP-1, α-tubulin, PMP70). For functional validation, pMT were rinsed with SHE buffer (0.25 M sucrose, 20 mM HEPES, 2 mM EGTA, 10 mM KCl, 1.5 mM MgCl_2_; pH 7.4). Complex I + III activity was measured by incubating 2 μg of mitochondria with 2.5 mM phosphate buffer (pH 7.5), 0.1% BSA, 0.3 mM KCN and 0.05 mM oxidized cytochrome c. After 2 min, 0.2 mM NADH was added, and absorbance at 550 nm was recorded for 20 min in kinetic mode using a Synergy HTX microplate reader (BioTek, USA). Complex IV activity was assessed using 2 μg of pMT incubated with 25 mM phosphate buffer (pH 7.0) and reduced cytochrome c. ATP synthesis capacity was measured by incubating 10 μg of pMT with 5 mM ADP for 45 min, followed by luminescence detection using CellTiter-Glo 2.0 reagent (Promega, USA). Citrate synthase activity was quantified using a commercial kit (MitoCheck; Cayman, USA), and heat-inactivated pMT (100°C, 20 min) served as a negative control.

### Cell culture

L6 rat myoblast (ATCC, Manassas, VA, USA) were cultured in Dulbecco’s Modified Eagle Medium (DMEM; Welgene Inc., Gyeongsan, Republic of Korea) supplemented with 10% fetal bovine serum (Gibco, USA) at 37°C in a humidified 5% CO_2_ atmosphere. Upon reaching confluence, cells were induced to differentiate in DMEM containing 2% horse serum (Gibco), with media replaced every 2 days. After 7 days of differentiation, muscle atrophy was induced by treating the cells with 1 μM dexamethasone for 24 h to establish an *in vitro* model [24, 25].

### Transfer of pMT to L6 cells

Mitochondrial transfer was performed based on a previously reported centrifugation-enhanced delivery [26]. To minimize donor-to-donor variability, pMT isolates from multiple healthy human donors were pooled prior to each experiment. This approach was supported by internal validation showing consistent mitochondrial yield and activity across donors (see Supplementary Figure S1). Isolated pMT was suspended in Dulbecco’s phosphate-buffered saline (DPBS; Welgene Inc.), and the suspension was gently added to tubes containing L6 cells (1 × 10^5^ cells per condition). The mitochondrial dose was standardized based on total protein amount per recipient cell number. The mixture was subjected to centrifugation at 1500 × *g* for 5 min at 4°C to facilitate mitochondrial internalization while preserving organelle integrity.

### Experimental procedures for assessing cell viability and mitochondrial function

After mitochondrial transfer, L6 cells were assessed for viability, ATP production, mitochondrial reactive oxygen species (ROS) and mitochondrial membrane potential (ΔΨm). Cells were seeded in 96-well plates and incubated at 37°C. Cell viability was evaluated using a WST-based EZ-CytoX assay kit (DoGenbio, Seoul, Republic of Korea), and ATP content was quantified using the CellTiter-Glo 2.0 reagent (Promega, USA) following the manufacturer’s protocol. Mitochondrial ROS levels were measured using MitoSOX Red (Invitrogen, USA). Cells were incubated with 5 µM dye for 10 min at 37°C, followed by PBS wash. Fluorescence was measured using a Synergy HTX microplate reader (BioTek, USA) at 530/25 nm excitation and 590/30 nm emission. Mitochondrial membrane potential was analyzed using tetramethyl rhodamine ethyl ester (TMRE; Invitrogen) and quantified by flow cytometry (CytoFLEX, Beckman Coulter, Brea, CA, USA).

### Research design and establishing an *in vivo* PMA model

A total of 11 male Sprague Dawley rats (12 weeks, 340–370 g) were randomly divided into three groups: the pMT group (*n* = 5; pMT injection after surgery), the surgery group (*n* = 3; no post-surgical treatment) and the control group (*n* = 3; no surgery). All rats were housed under standard laboratory conditions (23 ± 3°C, 55 ± 15% humidity, 12 h/12 h light/dark cycle) with *ad libitum* access to food and water. All procedures were approved by the Institutional Animal Care and Use Committee (IACUC No. 23-KE-0135, HLB bioStep Animal Center, Songdo, Republic of Korea). Group sizes were intentionally limited in consideration of ethical guidelines for animal research. The selected sample sizes (*n* = 3 for control and surgery-only groups; *n* = 5 for the pMT group) were informed by pilot experiments that validated the hemilaminectomy-induced muscle atrophy model and identified the 2-week endpoint as optimal for evaluation. Although formal power analysis was not performed, the observed within-group differences were statistically significant (*P* < 0.05) with a large effect size. Future studies should employ larger group sizes to ensure adequate statistical power and confirm reproducibility.

To induce paraspinal muscle atrophy, a previously established spinal surgery protocol was applied at T0 [27]. Under anesthesia, hemilaminectomy was performed and the left L5 spinal nerve was ligated using 6–0 silk sutures (Supplementary Figure S2). The laminas of L5 and L6, including the L5–L6 facet joint, were removed to simulate surgical muscle injury and denervation-induced disuse atrophy. At T1 (2 weeks after surgery), each group received its respective treatment. At T2 (2 weeks after T1), the animals were euthanized and paraspinal muscle tissues were harvested for analysis. Muscle appearance was also visually assessed following skin removal.

### Intramuscular pMT injection

Isolated pMT were injected into the left paraspinal muscles at the L4–L5 and L5–L6 levels using a 29-gauge syringe (25 μg of pMT in 100 μL of SHE buffer per site; total 50 μg per rat), as described in Queen’s University SOP 10-9-3 and in accordance with international animal care guidelines [28].

To ensure accurate targeting, the injection procedure was performed under fluoroscopic guidance and verified using C-arm imaging. The injection sites were localized by identifying the midpoints between the spinous processes of L4–L5 and L5–L6, and positioning the needle slightly lateral to the connecting line. The depth of the multifidus muscle was measured using MRI, and injections were administered accordingly (Supplementary Figure S3).

### Measuring cytokine concentrations

Plasma concentrations of inflammatory cytokines (IFN-γ, IL-17A, IL-1β, IL-6 and TNF-α) were measured at T2 (28 days after surgery). Blood samples were collected immediately prior to sacrifice, and plasma was separated by centrifugation at 10 000 × *g* for 10 min. The supernatants were analyzed using a multiplex cytokine assay kit (RECYTMAG-65K; Merck Millipore, USA) on a Luminex platform (Luminex, Austin, TX, USA), and concentrations were quantified using MasterPlex QT 2010 software (MiraiBio Inc., Tokyo, Japan).

### Confirmation of mitochondrial transplantation

Successful delivery of pMT to paraspinal muscle was confirmed by immunofluorescence staining. Muscle tissues were fixed in 4% paraformaldehyde, dehydrated, embedded in paraffin and sectioned into 4 μm-thick slices. After rehydration and PBS washing, sections were blocked with 5% normal horse serum (Vector Laboratories Inc., USA). Primary antibodies against Desmin (Abcam, Cambridge, UK; ab32362) and human mitochondria (Abcam, ab92824) were applied at 1:100 dilution and incubated overnight at 4°C. Following PBS rinsing, Alexa Fluor 488-conjugated goat anti-rabbit IgG and Alexa Fluor 594-conjugated goat anti-mouse IgG (Invitrogen) were applied at 1:300 dilution for 1 h in the dark at room temperature. Nuclei were counterstained with DAPI (Vector Laboratories), and sections were visualized using an Eclipse Ts2 inverted fluorescence microscope (Nikon, Japan).

### Pain threshold assessment

Mechanical pain sensitivity was assessed using the von Frey test at defined time points. Von Frey filaments (North Coast Medical, Morgan Hill, CA, USA) were applied to the plantar surface of the left hind paw to evaluate withdrawal responses. Each rat was tested three times, and the pain threshold was defined as the minimum force required to elicit a withdrawal reflex.

### Histological analysis

Histological evaluation was performed using hematoxylin and eosin (H&E) staining (Sigma–Aldrich, Burlington, MA, USA). Stained muscle sections were examined under an ECLIPSE E600 microscope (Nikon, Japan), and the cross-sectional area (CSA) of individual muscle fibers was measured. A total of 100 fibers were randomly selected from each muscle sample for quantification. CSA was estimated by manually drawing the region of interest using ImageJ (National Institutes of Health, Bethesda, MD, USA). Analysis reliability was confirmed through repeated measurements by two researchers.

### RNA sequencing

Total RNA was extracted from the multifidus tissues using the easy-spin total RNA extraction kit (iNtRON Biotechnology, Seoul, Republic of Korea). Paired-end sequencing was performed on an Illumina NovaSeq platform (Illumina, San Diego, CA, USA). Adapter trimming and quality filtering were conducted using Trimmomatic (v0.38). Processed reads were aligned to the Rattus norvegicus reference genome (rn6) using HISAT2 (v2.1.0) with Bowtie2 integration. Genome and annotation files were obtained from NCBI Genome Assembly and RefSeq. Aligned reads were sorted and indexed with SAMtools (v1.9), and transcript assembly and quantification were performed using StringTie (v2.1.3b).

Gene expression data are available in the Gene Expression Omnibus (GEO; accession number GSE267096). Differential gene expression analysis was conducted using edgeR (v3.40.2), with the exactTest function applied to raw count data. *P*-values were adjusted using the Benjamini–Hochberg method, and differentially expressed genes (DEGs) were defined as those with |fold change| ≥ 2 and *P *< 0.05.

Gene ontology (GO) enrichment analyses were conducted via g: Profiler (version e110_eg57_p18_4b54a898), classifying DEGs into cellular components (CC), molecular functions (MF) and biological processes (BP). The top 10 GO terms in each category were selected for further analysis. KEGG pathway analysis was performed using the DAVID online tool, and gene set enrichment analysis (GSEA) was conducted using GO terms as predefined gene sets to compare the pMT and surgery groups.

### Western blotting

To assess mitochondrial purity, pMT samples were heated at 95°C for 7 min in SDS-PAGE loading buffer (LPS Solution, Daejeon, Republic of Korea). Proteins were separated by SDS-PAGE and transferred onto PVDF membranes, which were then blocked with 5% bovine serum albumin for 1 hr, and incubated with the following primary antibodies: anti-COX IV (Cell Signaling Technology, Danvers, MA, USA; 4844s), anti-TOM20 (Santa Cruz Biotechnology Inc., Dallas, TX, USA; sc-17764), anti-p84 (Abcam; ab131268), anti-α-tubulin (Santa Cruz; sc-8035), anti-LAMP-1 (Santa Cruz; sc-20011) and anti-PMP70 (Abcam; ab3421). After washing, membranes were incubated with HRP-conjugated secondary antibodies (Santa Cruz; sc-516102 and sc-2357). Detection was performed using an Amersham ECL kit (Cytiva), and bands were visualized with a LAS-4000 system (Fuji, Japan).

To assess muscle tissue markers, lysates were prepared using RIPA buffer supplemented with a Halt protease and phosphatase inhibitor cocktail (Thermo Scientific). Protein concentrations were measured using a BCA assay. Primary antibodies used included: anti-GAPDH (Cell Signaling; 2118s), anti-Desmin (Abcam; ab32362), anti-myogenin (Santa Cruz; sc-12732), anti-AMPK (Cell Signaling; 2532s), anti-p-AMPK (Cell Signaling; 2535s), anti-AKT (Cell Signaling; 9272s), anti-p-AKT (Cell Signaling; 4060s), anti-FoxO3a (Cell Signaling; 2497s), anti-Bax (Santa Cruz; sc-7480), anti-Bcl-2 (Santa Cruz; sc-7382), anti-OXPHOS (Abcam; ab110413), anti-COX IV (Cell Signaling; 4844s) and anti-PGC1α (Novus Biologicals, Littleton, CO, USA; NBP1-04676). Band intensities were quantified using ImageJ. Other procedures followed the same protocol described above.

### Statistical analysis

Statistical analyses were performed using GraphPad Prism 5 software (GraphPad Software Inc., San Diego, CA, USA). For comparisons between two groups, Student’s *t*-test was used. For comparisons among three or more groups, one-way analysis of variance (ANOVA) followed by Tukey’s *post hoc* test was applied. Data are expressed as mean ± standard deviation (SD), and statistical significance was set at *P *< 0.05.

## Results

### Isolated human pMT display structural integrity, purity and preserved activity

**Figure 1.**
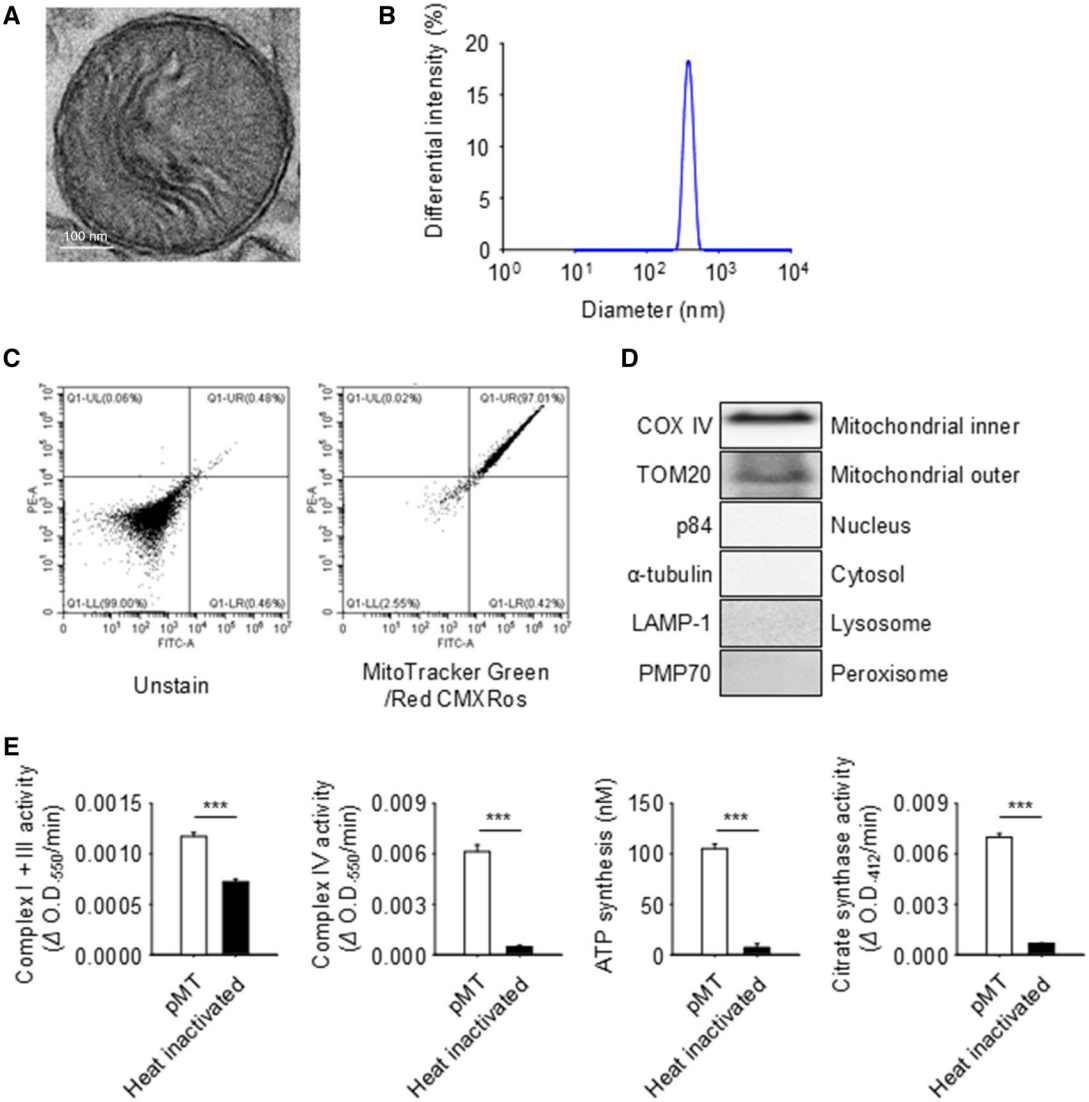
Quality control of isolated human pMT. (**A**) Transmission electron microscopy (TEM) image showing intact double membrane and cristae. Scale bar = 100 nm. (**B**) Size distribution of pMT measured by dynamic light scattering (DLS). (**C**) Flow cytometry analysis confirming mitochondrial identity using dual staining with MitoTracker Green and Mitotracker Red CMXRos. (**D**) Western blot analysis showing presence of mitochondrial markers (COX IV, TOM20) and absence of contamination markers: p84 (nuclear), α-tubulin (cytosolic), LAMP-1 (lysosomal), PMP70 (peroxisomal). (**E**) Functional assessment of mitochondrial activity via Complex I + III and Complex IV assays, ATP production and citrate synthase activity. Inactivated pMT (heated at 100°C for 20 min) served as negative controls. Data are presented as mean ± SD (*n* = 3); ***P < 0.001.

Transmission electron microscopy confirmed that the isolated human pMT retained typical mitochondrial architecture, including a well-defined double membrane and distinct cristae (Figure 1A). Size distribution analysis using dynamic light scattering (DLS) showed that the average pMT diameter was 383 ± 43.9 nm (Figure 1B). To verify the mitochondrial identity of the isolated particles, flow cytometry analysis was performed using dual labeling with MitoTracker Green and Mitotracker Red CMXRos. The results revealed a high proportion of MitoTracker^+^ events, confirming that the isolated particles were indeed mitochondria (Figure 1C). Western blot analysis further validated the purity of the isolated pMT, with the presence of mitochondrial markers COX IV and TOM20, and absence of non-mitochondrial markers such as p84 (nucleus), α-tubulin (cytosol), LAMP-1 (lysosome) and PMP70 (peroxisome) (Figure 1D). To evaluate functional integrity, we assessed respiratory enzyme activities including Complex I + III and Complex IV, as well as ATP synthesis and citrate synthase activity. All parameters were significantly preserved in intact pMT compared to heat-inactivated controls, confirming their bioenergetic competence (Figure 1E).

Furthermore, to assess donor variability, we examined pMT yield and ATP production across diverse age groups and individuals with stable chronic conditions. No statistically significant differences were observed (Supplementary Figure S1A–D), suggesting that pMT quality is consistent regardless of donor age or health status. These results highlight the feasibility of utilizing autologous pMT as a stable therapeutic resource.

### Mitochondrial transfer improves cell viability and mitochondrial function in Dexa-induced L6 cells

**Figure 2.**
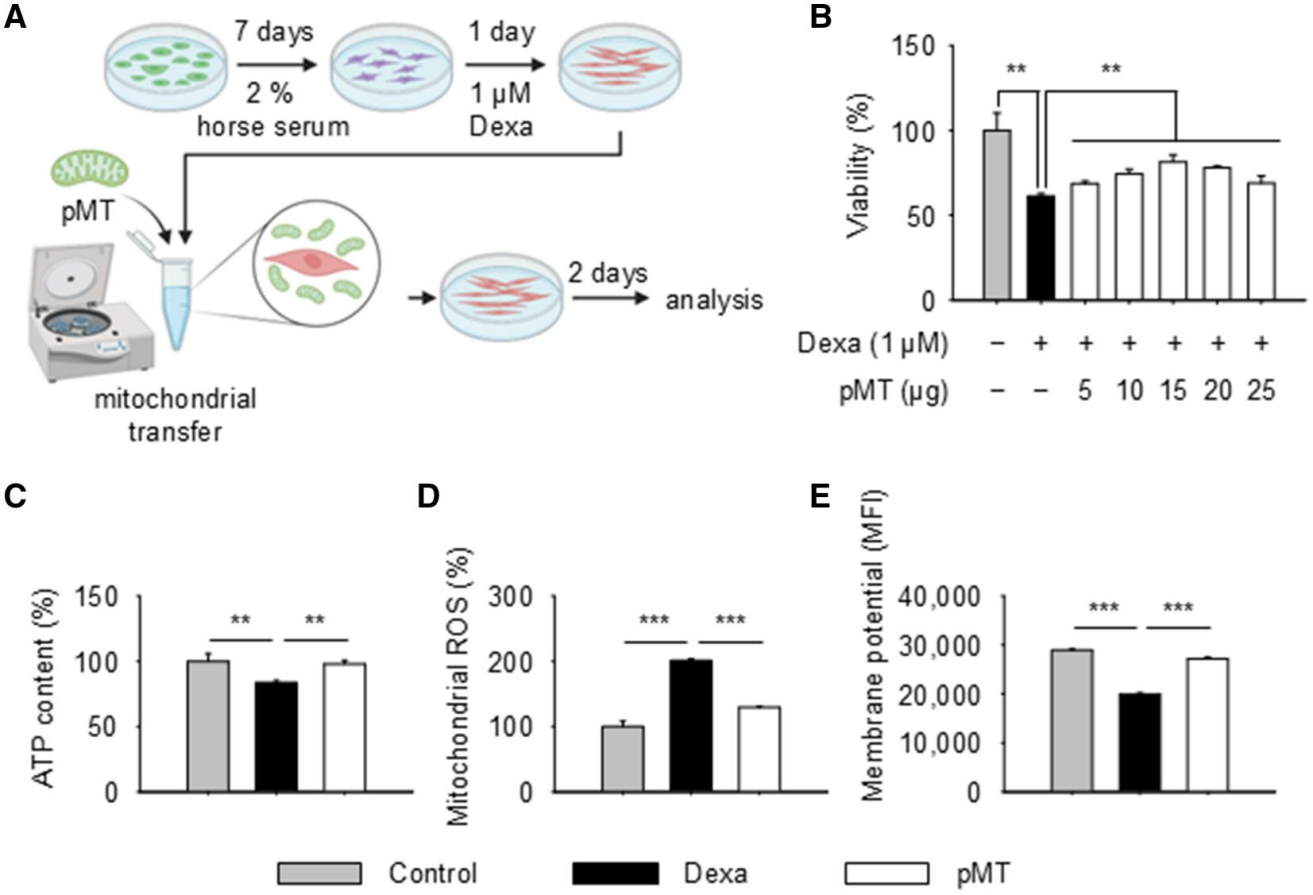
pMT transfer restores cell viability and mitochondrial function in Dexa-induced atrophied L6 cells. (**A**) Experimental scheme of *in vitro* atrophy model using L6 myotubes treated with dexamethasone (Dexa). (**B**) Cell viability after pMT treatment at various doses; 15 μg dose produced the most pronounced improvement. (**C–E**) Mitochondrial function was evaluated by ATP content (**C**), mitochondrial ROS (**D**) and membrane potential (**E**). Data are presented as mean ± SD (*n* = 3), **P < 0.01, ***P < 0.001.

Before applying pMT therapy in animal models, we first tested its effects on cellular function using an *in vitro* atrophy model. Dexamethasone (Dexa) induced muscle atrophy in differentiated L6 myotubes (Figure 2A). pMT-treated cells showed significantly improved viability compared to the Dexa-only group, with the best response observed at 15 μg (Figure 2B). Based on this, we used 15 μg as the standard dose for further tests. Mitochondrial function was also assessed through ATP content, ROS levels and membrane potential. All indicators showed improvement following pMT transfer (Figure 2C–E), supporting its potential for reversing Dexa-induced dysfunction.

### Establishment of rat model of PMA via spinal surgery and the therapeutic efficacy of pMT

**Figure 3.**
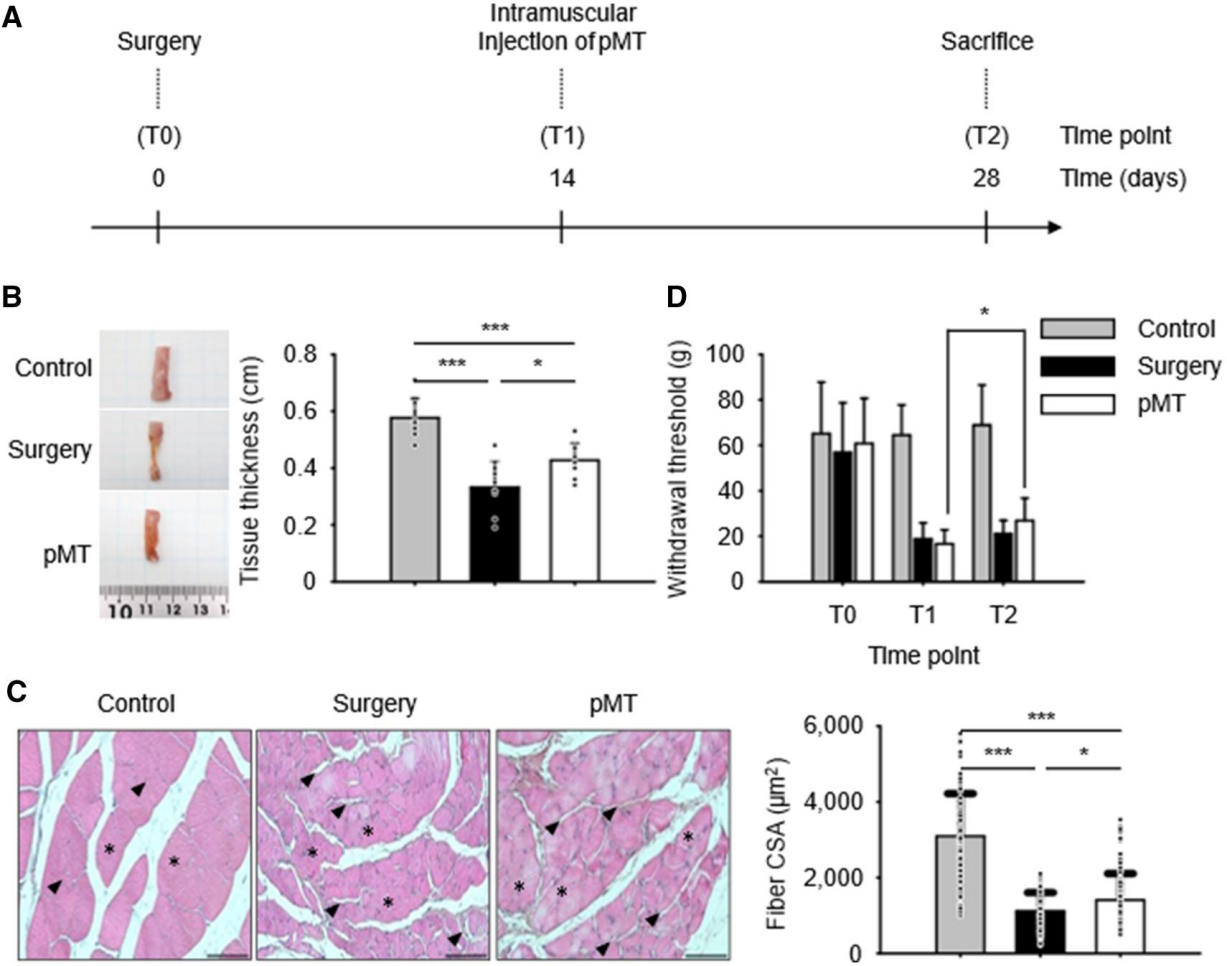
pMT transplantation alleviates muscle atrophy and pain in a rat model of spinal surgery-induced PMA. (**A**) Schematic timeline of the experimental procedure. Rats underwent spinal surgery at T0 (Day 0), followed by intramuscular injection of pMT at T1 (Day 14). Tissues were collected at T2 (Day 28) for histological and molecular analysis. (**B**) Representative images and quantification of paraspinal muscle thickness. (**C**) H&E staining and analysis of muscle fiber cross-sectional area (CSA). For each biological replicate (*n* = 3 for control and surgery-only; *n* = 5 for pMT), ≥100 fibers per animal were measured to assess fiber size distribution (technical replicates). Black arrowheads (▲) indicate inter-fiber spacing—noticeably widened in the surgery-only group and reduced in the pMT group. Black asterisks (*) denote representative muscle fibers, which appear irregular in the surgery-only group and show improved morphology in the pMT group. Annotated version of these images is provided in Supplementary Figure 6. All images were acquired at 100 × magnification; scale bar = 100 μm. (**D**) Mechanical withdrawal threshold measured by von Frey test at baseline (T0), postsurgery (T1) and post-treatment (T2). The pMT group exhibited a significant intra-group improvement from T1 to T2 (*P *< 0.05), while no improvement was observed in the surgery-only group. For each rat, withdrawal thresholds were measured in triplicate at each time point. Data are presented as mean ± SD for each group (*n* = 3 for control and surgery-only, *n* = 5 for pMT). *P < 0.05, ***P < 0.001.

To evaluate the therapeutic effects of pMT *in vivo*, we established a rat model of PMA through spinal surgery. The overall experimental timeline is shown in Figure 3A. At the endpoint (T2), macroscopic assessment revealed noticeable muscle loss on the operated side (Supplementary Figure S4A). This clarification improves the accuracy and specificity of our description. Interestingly, there was no significant change in body weight between groups, suggesting that the atrophy was localized and not due to systemic effects (Supplementary Figure S4B). Additionally, plasma cytokine levels remained unchanged, indicating that the surgery did not trigger systemic inflammation (Supplementary Figure S4C).

After confirming successful PMA induction, we examined the effect of pMT treatment. In the pMT-injected group, immunofluorescence staining confirmed the presence of human mitochondria within the paraspinal muscle tissue (Supplementary Figure S5). Gross morphology and histological analysis showed muscle shrinkage in the surgery group, whereas the pMT group displayed thicker muscles and improved fiber integrity (Figure 3B and C). Quantification of muscle CSA also demonstrated a significant improvement in the pMT group.

To assess whether pMT also affected pain sensitivity, we conducted von Frey filament testing. Withdrawal thresholds decreased after surgery, consistent with increased mechanical hypersensitivity. However, rats receiving pMT treatment showed significantly higher thresholds, indicating attenuated mechanical hypersensitivity (Figure 3D).

### RNA sequencing analysis for functional prediction of DEGs

**Figure 4.**
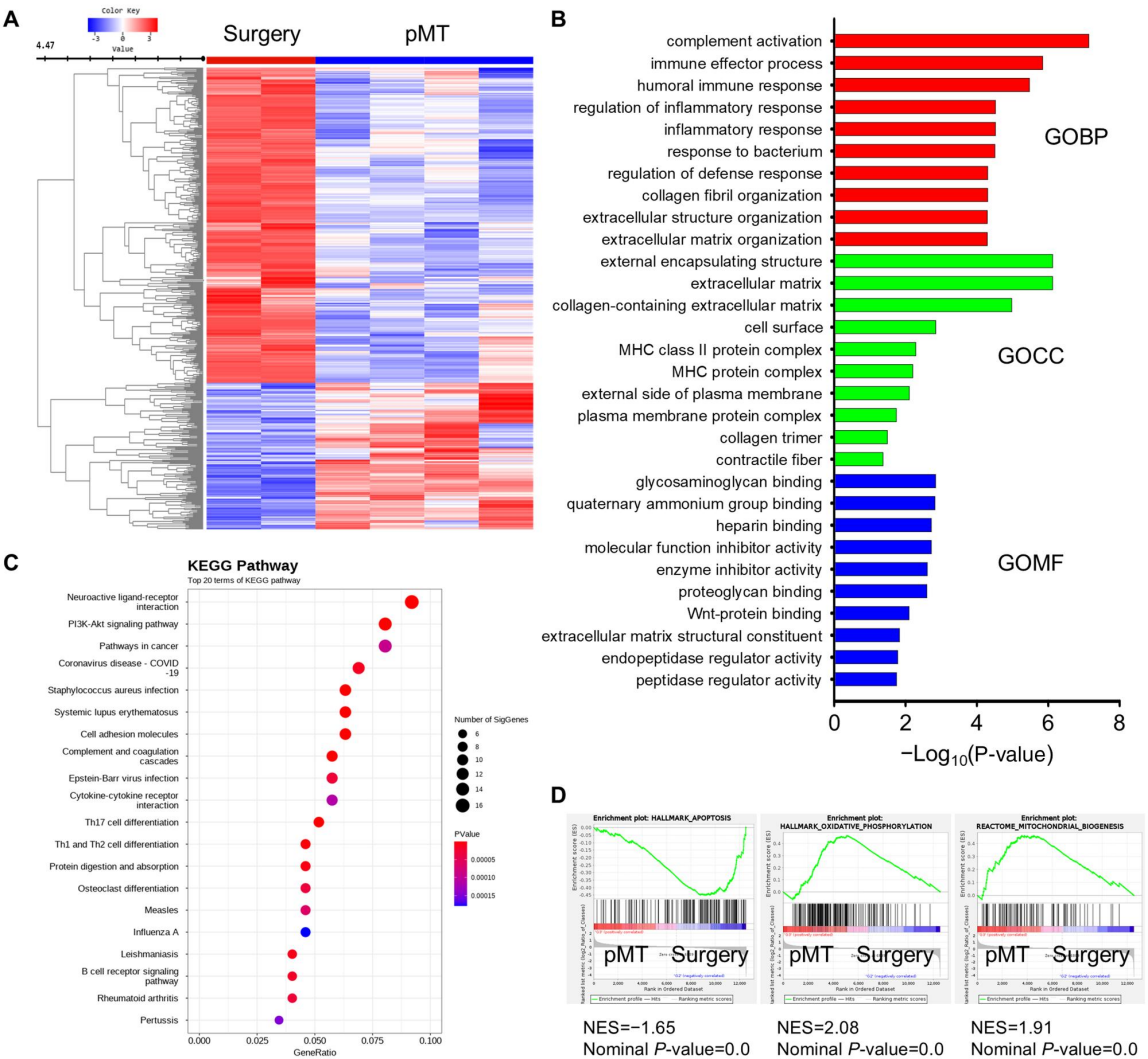
RNA Sequencing reveals transcriptional changes induced by pMT transplantation. (**A**) Heatmap showing differentially expressed genes (DEGs) between surgery and pMT groups. (**B**) Gene ontology (GO) analysis of DEGs: top 10 terms in biological processes (BP), cellular components (CC) and molecular functions (MF). (**C**) KEGG pathway enrichment analysis showing top 20 enriched pathways. (**D**) Gene set enrichment analysis (GSEA) indicating suppression of apoptosis and upregulation of oxidative phosphorylation (OXPHOS) and mitochondrial biogenesis in the pMT group.

To understand how pMT transplantation affects gene expression in PMA, we performed RNA sequencing on multifidus muscle samples from the surgery and pMT groups. A total of 510 differentially expressed genes (DEGs) were identified, with 162 genes upregulated and 348 downregulated in the pMT group (Figure 4A). To explore the biological significance of these changes, we conducted Gene Ontology (GO) and KEGG pathway enrichment analyses. GO terms were enriched across 13 cellular components, 223 biological processes and 15 molecular function categories (Figure 4B). KEGG analysis revealed that the DEGs were involved in 49 signaling pathways (Figure 4C).

Gene set enrichment analysis (GSEA) further showed that apoptosis-related gene sets were suppressed in the pMT group, while pathways related to oxidative phosphorylation (OXPHOS) and mitochondrial biogenesis were upregulated (Figure 4D). These findings suggest that pMT transplantation may exert its therapeutic effects by modulating mitochondrial activity and reducing cell death.

### pMT transplantation inhibited apoptosis through mitochondrial biogenesis

**Figure 5.**
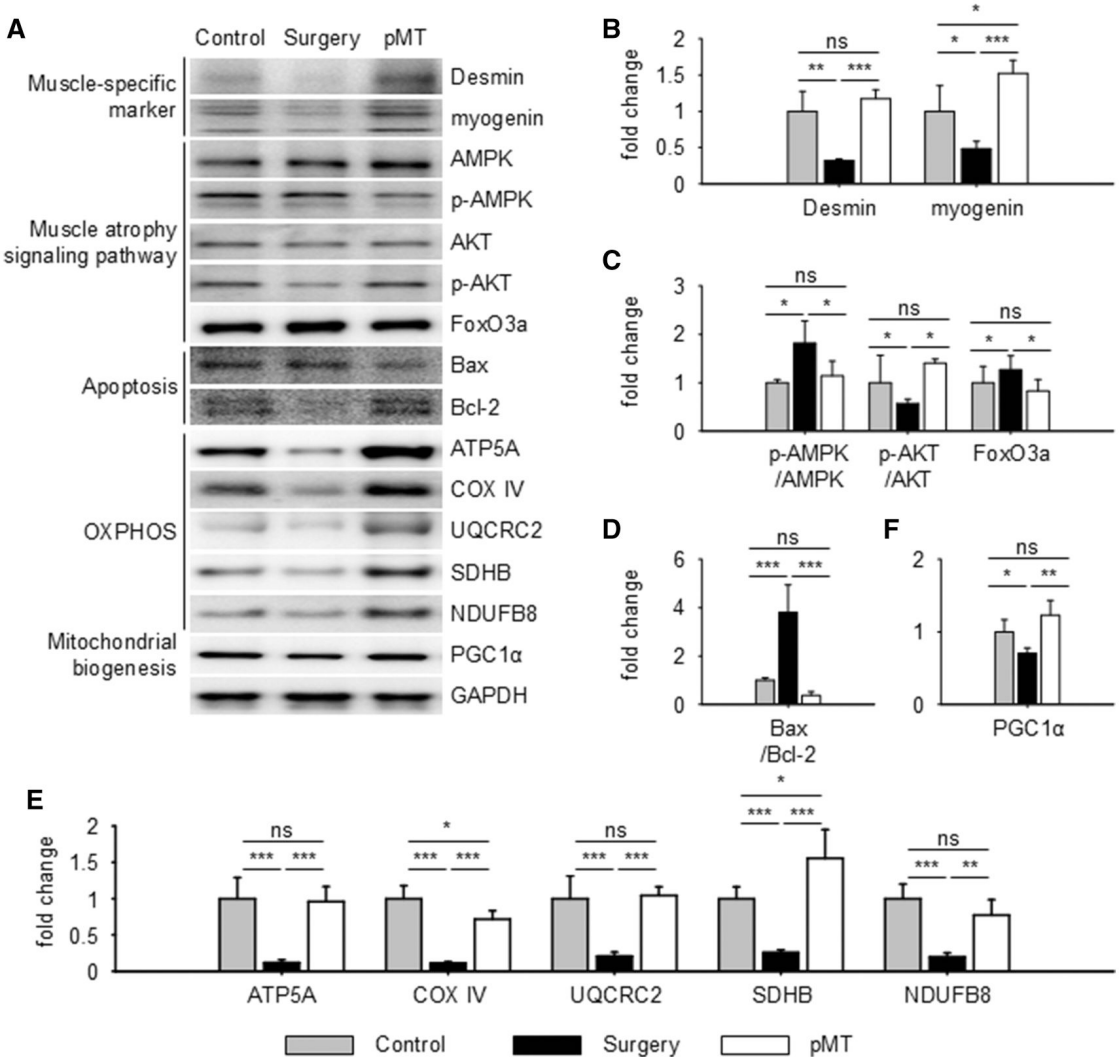
pMT modulates mitochondrial biogenesis, signaling pathways and apoptosis at the protein level. (**A**) Western blot images of proteins related to muscle structure, signaling, apoptosis and mitochondrial function. (**B**) Muscle-specific markers (Desmin, myogenin). (**C**) AMPK/AKT/FoxO3a pathway proteins. (**D**) Apoptosis-related markers (Bax, Bcl-2). (**E)** OXPHOS complex I–V proteins. (**F**) Mitochondrial biogenesis marker PGC1α. Western blot quantification was performed using independently obtained muscle samples from biological replicates (*n* = 3 for control and surgery-only; *n* = 5 for pMT). Each blot was independently repeated at least once to ensure reproducibility. Data are presented as mean ± SD; *P < 0.05, **P < 0.01, ***P < 0.001. ns (not significant).

Based on the transcriptomic data suggesting that pMT modulates mitochondrial and apoptosis-related pathways, we next assessed whether these changes were reflected at the protein level (Figure 5A). We first examined Desmin and myogenin, markers of muscle structure and regeneration. Both were significantly reduced in the surgery group but were restored following pMT transplantation (Figure 5B). Since muscle atrophy is associated with impaired protein turnover, we examined the AMPK/AKT/FoxO3a signaling axis. After surgery, AMPK and FoxO3a levels increased while AKT decreased, consistent with muscle atrophy. pMT treatment reversed these changes, suggesting a role in promoting anabolic signaling and suppressing atrophy (Figure 5C). Apoptosis markers were also evaluated. Bax was upregulated and Bcl-2 was downregulated after surgery, indicating increased apoptosis. These effects were reversed by pMT treatment (Figure 5D). To assess mitochondrial function, we measured the expression of oxidative phosphorylation (OXPHOS) complexes I–V, which were suppressed by surgery but recovered after pMT treatment (Figure 5E). In addition, the expression of PGC1α, a master regulator of mitochondrial biogenesis, was restored in the pMT group (Figure 5F).

## Discussion

Paraspinal muscle atrophy is a common and clinically significant complication after spinal surgery. It leads to structural deterioration and compromised spinal stability through multiple pathological mechanisms [29]. Although minimally invasive surgical techniques have been developed to reduce PMA, its occurrence even after single-level discectomies suggests that current strategies remain insufficient [30, 31]. These findings underscore the need for regenerative approaches that go beyond conventional interventions such as exercise. In this study, we demonstrated that transplantation of pMT effectively mitigated PMA by increasing muscle fiber cross-sectional area, restoring mitochondrial function and reducing pain sensitivity. These results provide compelling evidence for pMT as a promising regenerative modality to overcome the limitations of existing treatments.

The observed increase in muscle CSA highlights the essential role of mitochondrial function in reversing PMA-related muscle atrophy [32]. Mitochondrial dysfunction—characterized by impaired ATP production, elevated reactive oxygen species (ROS) and activation of proteolytic pathways—is a key contributor to muscle degeneration [33]. Transplantation of pMT effectively restored mitochondrial bioenergetics, as evidenced by enhanced oxidative phosphorylation (OXPHOS) activity and increased ATP levels [13, 34]. Since OXPHOS is central to sustaining protein synthesis, cellular repair and regeneration, its recovery is crucial in the context of muscle preservation [35]. In PMA-affected tissues, disrupted OXPHOS reduces energy availability and promotes excessive ROS generation, which exacerbates oxidative damage and accelerates protein breakdown [36]. By stabilizing mitochondrial membranes and modulating ROS levels, pMT alleviate oxidative stress and help preserve mitochondrial integrity [18, 37], supporting cellular resilience and regeneration. In addition to restoring bioenergetics and reducing apoptosis, transplanted mitochondria may also buffer cytosolic Ca^2+^ levels [38]. This could prevent Ca^2+^-dependent activation of proteases and the opening of the mitochondrial permeability transition pore (mPTP), thereby preserving mitochondrial structure and function. A similar mechanism has been reported in dystrophin-deficient muscles [39]. Although we did not measure Ca^2+^ homeostasis directly in this study, this hypothesis warrants future investigation using calcium-sensitive probes and live-cell imaging.

The upregulation of myogenin and Desmin—key markers of muscle fiber development and structural integrity—indicates that muscle regeneration was successfully initiated following pMT transplantation [40, 41]. These changes were accompanied by alterations in gene and protein expression profiles, as well as increased fiber cross-sectional area, likely mediated by modulation of the AMPK/AKT/FoxO3a signaling axis. AMPK activity is regulated by the intracellular ATP/AMP and ATP/ADP ratios [42, 43]; thus, reduced ATP levels due to muscle damage can activate AMPK and promote catabolic signaling that contributes to atrophy. By restoring ATP levels, pMT may have attenuated AMPK activation while promoting AKT signaling, thereby supporting anabolic processes. In addition, pMT treatment suppressed pro-apoptotic signals and enhanced anti-apoptotic pathways, further contributing to muscle preservation under conditions of mitochondrial dysfunction.

The increased expression of PGC1α observed in this study indicates enhanced mitochondrial biogenesis, which may lead to improved mitochondrial density and cellular resilience [44]. PGC1α is a key regulator of mitochondrial biogenesis and plays an essential role in promoting muscle adaptation under oxidative and metabolic stress conditions. Previous studies have shown that PGC1α-mediated pathways contribute to the structural and functional recovery of mitochondria in stressed tissues. Therefore, the upregulation of PGC1α following pMT transplantation not only reflects restored mitochondrial function but also suggests a greater potential for long-term muscle regeneration and adaptation [45].

*In vitro* experiments confirmed the beneficial effects of pMT, particularly at a dose of 15 μg, indicating a saturation point beyond which no additional therapeutic advantage is observed (Figure 2B). The lack of additional benefit at higher doses suggests a saturation point in therapeutic efficacy, potentially reflecting either a plateau in bioenergetic restoration or cellular regulatory limits on exogenous mitochondrial processing.

Although no statistically significant difference was observed between the surgery-only and pMT groups at T2, the pMT group showed a significant intra-group improvement from T1 to T2 (*P *< 0.05), while the surgery-only group remained unchanged. This suggests that pMT promoted functional recovery over time. Furthermore, structural improvements observed in the pMT group (Figure 3C) support the therapeutic potential of pMT, which may not be fully reflected in behavioral assays with small sample sizes.

Importantly, despite their xenogeneic origin, intramuscular injection of human pMT into rats did not elicit a systemic immune response, as evidenced by stable levels of pro-inflammatory cytokines (TNF-α, IL-6, IL-1β, IFN-γ, IL-17A) in plasma (Supplementary Figure S4). Similarly, *in vitro* studies with LPS-stimulated human macrophage-like THP-1 cells showed no transcriptional or protein-level upregulation of inflammatory cytokines upon exposure to human-derived pMT (data not shown). These results suggest that isolated, membrane-intact pMT exhibit minimal immunogenicity, even though mitochondria contain damage-associated molecular patterns (DAMPs) such as mtDNA and cardiolipin. This low immunogenic profile is supported by previous studies. Ramirez et al. reported that both syngeneic and allogeneic mitochondria failed to elicit allo-reactive immune responses in recipient tissues, supporting the concept that mitochondria may be immunologically privileged organelles [46]. Similarly, other reports have shown minimal alloreactivity and innate immune activation following mitochondrial transplantation, particularly when mitochondrial membrane integrity is preserved [46, 47]. Nevertheless, long-term and repeated-dose studies are warranted to comprehensively evaluate the safety profile of pMT-based therapies.

The presence of transplanted mitochondria in recipient tissues is a critical factor influencing their therapeutic efficacy. In this study, only a small number of transplanted pMT were detectable two weeks after injection (Supplementary Figure S5), suggesting rapid clearance from the tissue. This observation aligns with our prior findings in allogeneic mitochondrial delivery to natural killer cells, where mitochondria were similarly cleared within two weeks. However, the literature remains divided: another group has detected exogenous mitochondria in recipient cells for periods extending up to 28 days or more, depending on delivery method and tissue context [16], others—including our previous work [48]—suggest that most exogenous mitochondria are cleared within approximately 2 weeks [49]. This variability likely reflects the complex interplay of delivery vehicles, host immune responses, mitophagy activity and fusion efficiency.

Rapid depolarization of mitochondrial membrane potential, particularly in calcium-rich extracellular environments, may trigger mitophagy via pathways such as PINK1–Parkin, accelerating degradation. Although mitophagy activation was not directly assessed, our independent biodistribution analysis revealed that exogenous mitochondrial DNA declined rapidly within 3 days, suggesting possible involvement of mitophagy or immune-mediated removal. In parallel, our recent study in a CLI model confirmed that mitochondria could enter damaged muscle fibers and partially integrate with the host network [50]. These findings imply that only a subset of injected mitochondria may persist, while the remainder is selectively eliminated. Elucidating the balance between clearance and integration will be key to optimizing therapeutic strategies.

Although our study did not directly assess mitochondrial fusion, supporting evidence from our recent work in a critical limb ischemia model showed the presence of transplanted mitochondria inside muscle fibers via transmission electron microscopy (TEM) [50], suggesting structural incorporation into recipient cells. These findings raise the possibility that, under appropriate conditions, pMT may engage in functional integration rather than acting solely via transient paracrine effects. In parallel, our previous studies including ultrastructural TEM analysis have confirmed intracellular localization of exogenous mitochondria in ischemic muscle tissue following systemic administration, supporting their internalization capacity and therapeutic relevance in different contexts. This further underscores the translational applicability of our pMT-based platform.

To further investigate mitochondrial persistence, our previous study tracked donor mitochondria in NK cells, revealing signal loss over several days via mtDNA qPCR and fluorescence imaging [50]. In addition, a recent GLP-compliant biodistribution study using tendon injection and RT-PCR quantification showed a steep decline in mitochondrial DNA by Day 3 and minimal levels by week 4 (*data not shown*). These findings underscore the transient nature of mitochondrial persistence and highlight the importance of optimizing dosing frequency in future therapeutic applications. Future studies are warranted to clarify the extent and duration of such integration and to identify the signaling pathways that mediate the observed regenerative outcomes.

Our findings demonstrate the regenerative potential of pMT transplantation in mitigating paraspinal muscle atrophy by restoring mitochondrial function and promoting structural muscle recovery. However, several limitations should be acknowledged. First, the small group sizes (*n* = 3 ∼ 5) may limit the reliability of our findings and reduce the ability to detect subtle differences between groups. Future studies with larger sample sizes are warranted to validate the therapeutic effects of pMT and strengthen the reproducibility of the results. Second, although a small portion of pMT was still detectable two weeks after injection, we did not examine earlier time points or quantify residual levels relative to the initial dose. Future studies are needed to track mitochondrial clearance dynamics using quantitative and time-resolved approaches. Third, the present study assessed only a single administration of pMT, leaving the safety and efficacy of repeated dosing unexamined. Given that we administered the maximum feasible dose (50 μg per rat) under resource and safety constraints, the optimal *in vivo* dosing of pMT remains to be established. Future dose–response studies are needed to determine the minimal effective dose and balance therapeutic efficacy with safety. Moreover, given the transient presence of transplanted pMT, determining the duration of sustained functional benefits and the necessity of repeated dosing remains a critical issue. In the present study, assessments of muscle fiber morphology and pain sensitivity were limited to two weeks post-injection, which coincided with the near-complete clearance of transplanted pMT. Thus, it remains unclear whether the observed improvements persist beyond the disappearance of exogenous mitochondria. Future studies should assess long-term outcomes and evaluate whether repeated administrations enhance or prolong therapeutic efficacy. Finally, we did not directly evaluate neuromuscular junction (NMJ) integrity or reinnervation. Future studies will include immunofluorescence analyses of postsynaptic acetylcholine receptors and presynaptic markers (e.g. synaptophysin, neurofilament) to assess NMJ preservation and functional restoration. Incorporating these endpoints will be critical to determine whether pMT therapy promotes not only myofiber regeneration but also neuromuscular reconnection.

While centrifugation was used to enhance mitochondrial uptake *in vitro*, *in vivo* uptake appears to occur under pathological conditions without physical forcing. In our recent CLI model study, mitochondria were internalized by ischemic muscle tissue without centrifugation [50]. This supports prior findings that cellular stress enhances mitochondrial entry via macropinocytosis or similar pathways. Notably, Kesner et al. reported that this process was blocked by heparan sulfate analogs, indicating receptor-mediated uptake [47]. Future studies are needed to quantify internalization efficiency and delineate the precise mechanisms.

This study represents the first investigation to demonstrate the therapeutic potential of pMT transplantation for PMA. Moreover, although our initial protocol yielded ∼2 µg of pMT protein per 1 mL of plasma (corresponding to ∼60 µg from each donor), recent improvements have increased the yield up to fivefold. Further characterization—such as nanoparticle tracking analysis—to determine the absolute number of mitochondrial particles and recovery efficiency would be valuable in optimizing and adapting this method for clinical applications. Future research should aim to further elucidate the underlying mechanisms and expand the application of pMT, including its integration with stem cell-based strategies.

In particular, combining pMT with mesenchymal stem cells may enhance regenerative efficacy by leveraging both mitochondrial replenishment and stem cell-mediated paracrine signaling. These strategies will contribute to the development of more effective and clinically translatable treatments for muscle atrophy following spinal surgery. Future studies should also investigate the safety and efficacy of repeated pMT administrations or encapsulated delivery systems to enhance therapeutic sustainability and overcome rapid mitochondrial clearance.

## Supplementary Material

rbaf090_Supplementary_Data
