# Supplementary material for: Plasma-derived mitochondrial transplantation attenuates paraspinal muscle atrophy following spinal surgery
Source: Regen Biomater. 2025 Aug 21;12:rbaf090. doi: 10.1093/rb/rbaf090 (PMC12449618; doi:10.1093/rb/rbaf090)
Supplement: rbaf090_Supplementary_Data [file rbaf090_supplementary_data.zip › Revised Supplementary figure legend_IH Lim et al.docx]

**Plasma-derived Mitochondrial Transplantation Attenuates Paraspinal Muscle Atrophy Following Spinal Surgery**

Ikhyun Lim^1,2,#^, Seong-Hoon Kim^1,#^, Mi Jin Kim^3^, Chang-Koo Yun^3^,

Kyunghoon Min^2*^, Yong-Soo Choi^1,3,4**^

^1^Department of Bioconvergence Science, Graduate School, CHA University, Seongnam 13488, Republic of Korea

^2^Department of Rehabilitation Medicine, CHA Bundang Medical Center, CHA University School of Medicine, Seongnam 13496, Republic of Korea

^3^Department of Biotechnology, CHA University, Seongnam 13488, Republic of Korea

^4^Department of Life Science, Graduate School, CHA University, Seongnam 13488, Republic of Korea

# These authors contributed equally to this work

Corresponding authors:

* minkh@chamc.co.kr; Tel.: (+82) 31-780-1892 (K. Min)

** yschoi@cha.ac.kr; Tel.: (+82) 31-881-7125 (Y.-S. Choi)


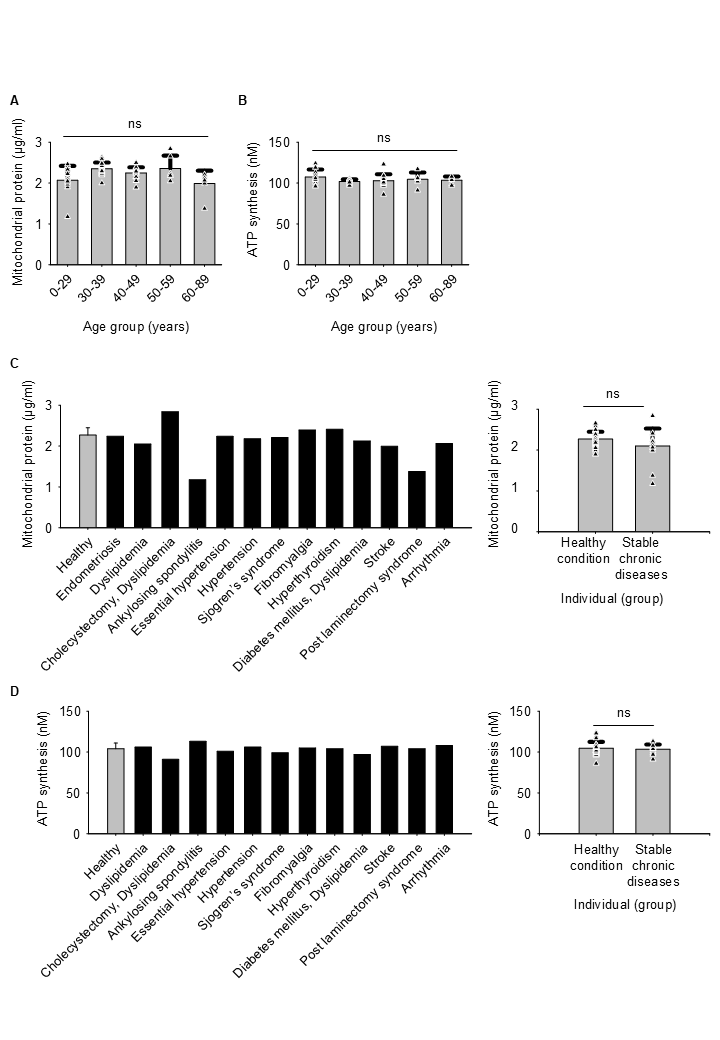


**Supplemental Figure 1.** Assessment of pMT quantity and ATP synthesis across different ages and health statuses. (A) pMT protein yield and (B) ATP synthesis rate analyzed across age groups: 20s (n = 11), 30s (n = 18), 40s (n = 17), 50s (n = 6), and 60s (n = 6). (C) pMT protein yield and (D) ATP synthesis rate compared between healthy individuals (n = 45) and those with stable chronic disease (n = 12-13). Data are presented as the mean ± standard deviation. ns (not significant).

**
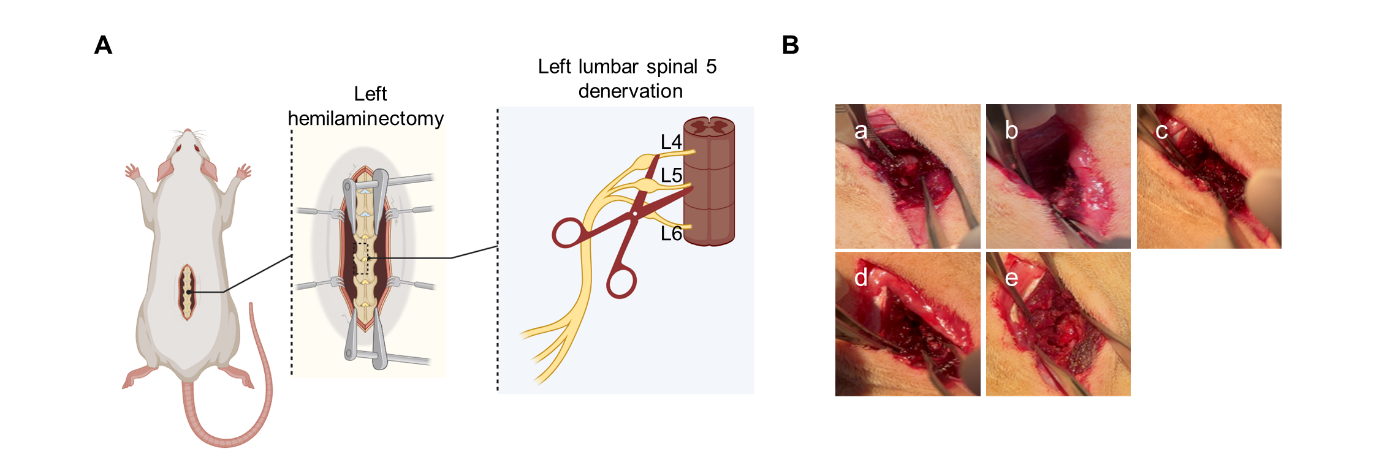
**

**Supplemental Figure 2.** Verification of an in vivo spinal surgery model. (A) Schematic illustration of the left hemilaminectomy and L5 spinal nerve denervation procedure. (B) Step-by-step photographs of the surgical procedure: (a) pre-surgery image showing the exposed bone; (b) Post-hemilaminectomy image revealing the spinal cord; (c) L5 nerve tied with 6-0 black silk; (d) Nerve severed proximal to the tied point; (e) Final post-surgery view.


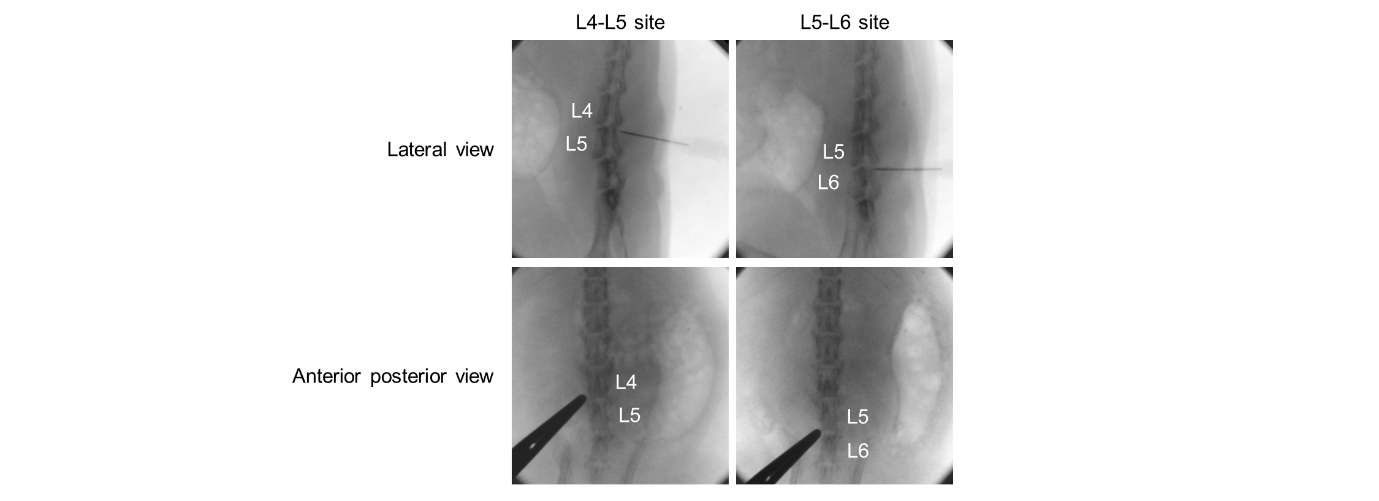


**Supplemental Figure 3.** Precise injection guided by C-arm imaging. An injection at a depth of 9 mm was administered into the left paraspinal muscle adjacent to the L4–L5 and L5–L6 spinous processes under C-arm guidance. Both lateral and anteroposterior views illustrate the needle position during the procedure.

**
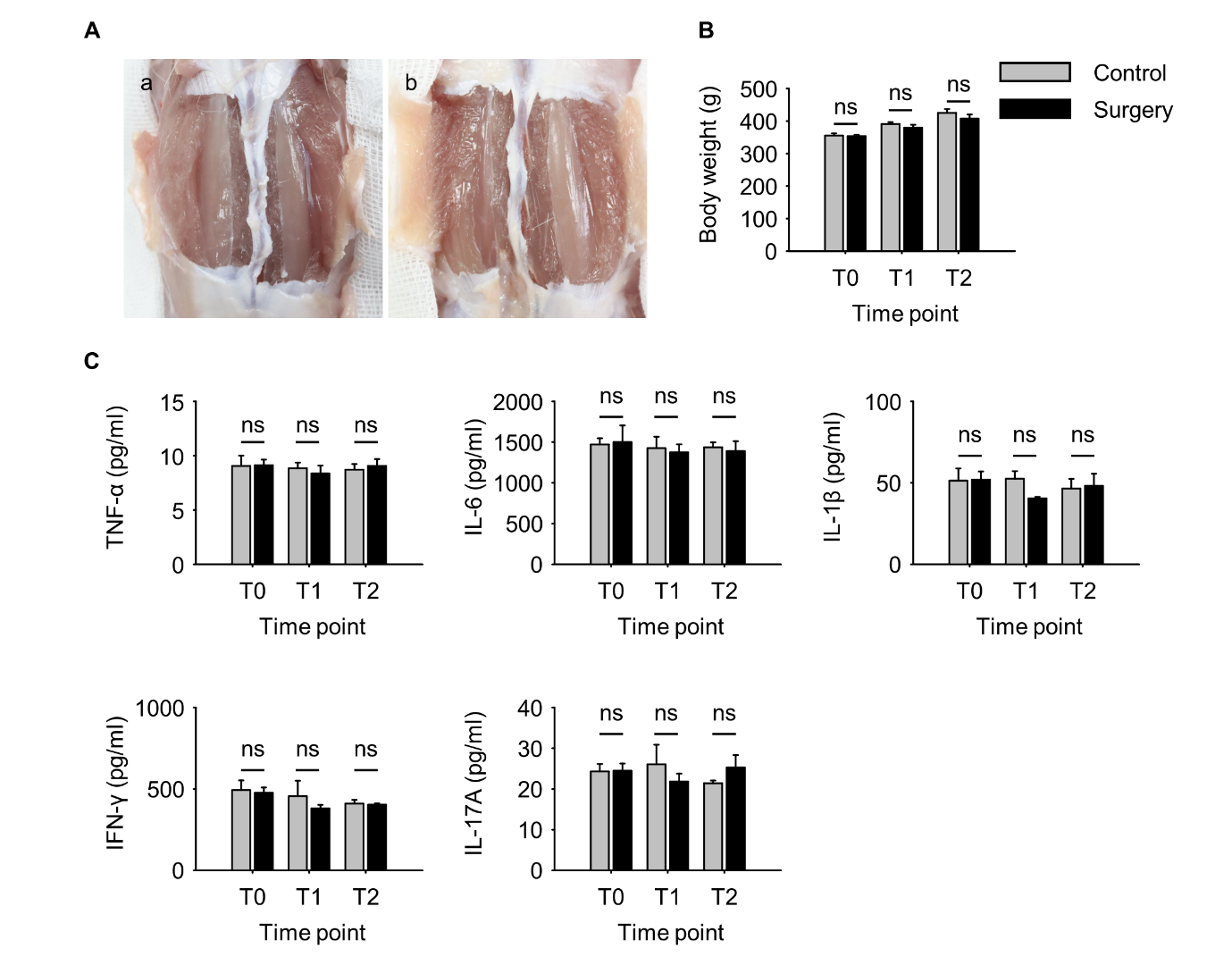
**

**Supplemental Figure 4.** (A) Post-mortem images of paraspinal tissues: (a) Control group without nerve severance; (b) Surgery group with nerve severance. (B) Body weight changes in both control and surgery groups at three time points: T0 (pre-surgery), T1 (two weeks post-surgery), and T2 (two weeks after T1). (C) Plasma cytokine analysis of TNF-α, IL-6, IL-1β, IFN-γ, and IL-17A in the control and surgery groups at T0, T1, and T2, obtained via multiplex analysis. Data are presented as the mean ± standard deviation. ns (not significant).


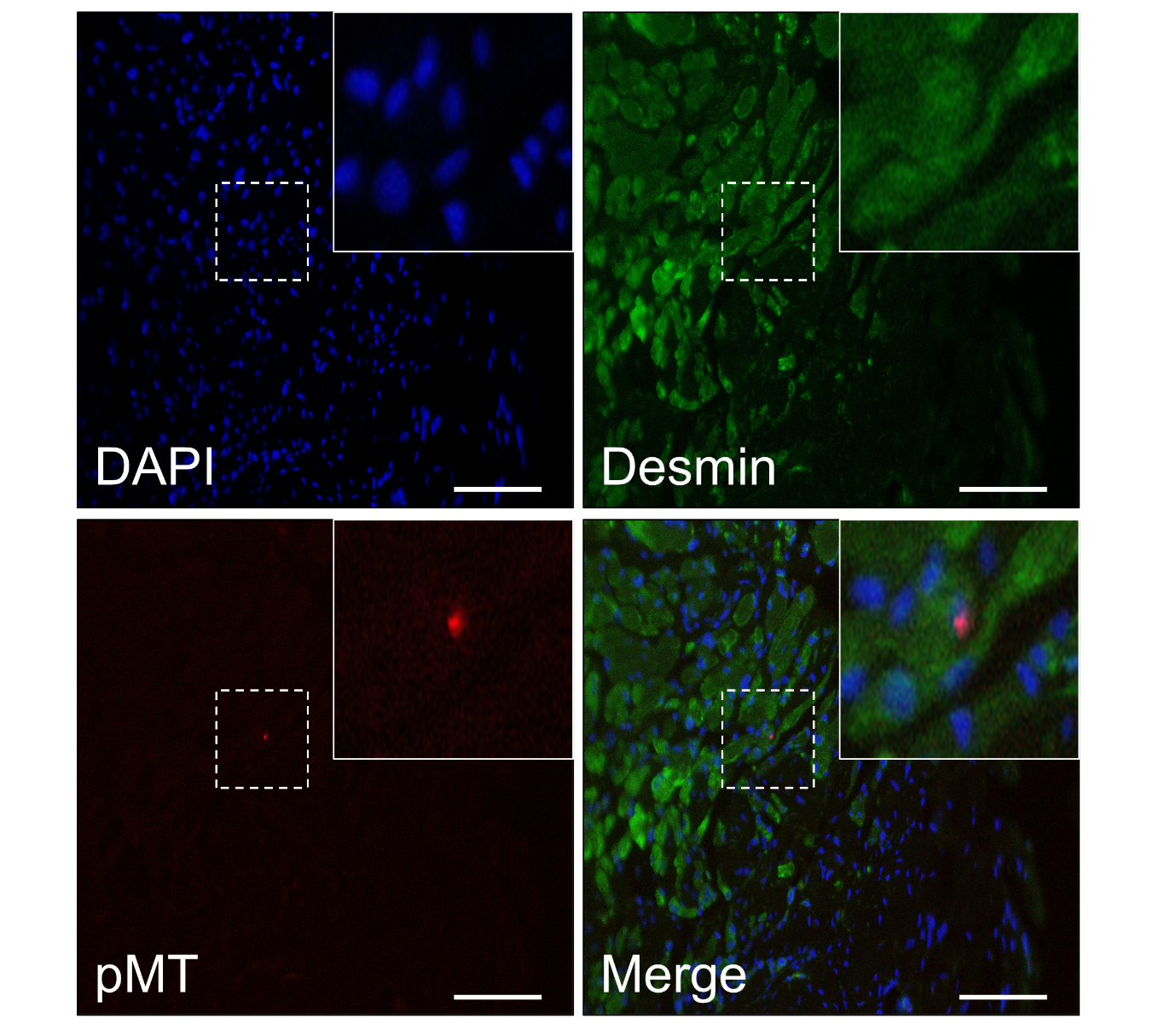


**Supplemental Figure 5.** Immunofluorescence analysis of transplanted human mitochondria in the paraspinal muscle 4 weeks post-transplantation. Images show human mitochondria (red) and Desmin (green). Scale bar = 100 μm.


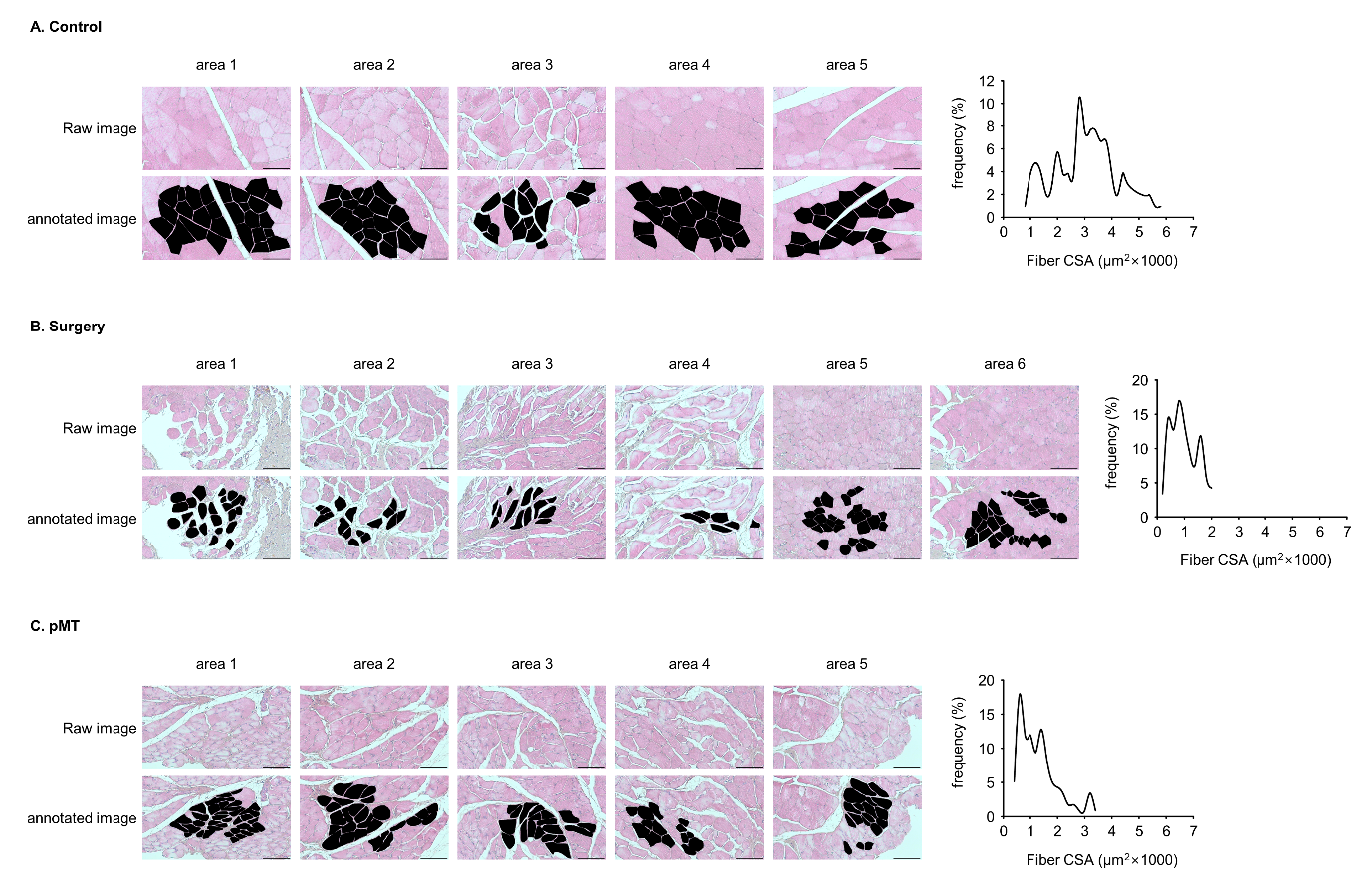


**Supplemental Figure 6.** Muscle sections (top panels) and annotated fiber segmentation (bottom panels) with fiber size distribution histograms. Each annotated image highlights individual muscle fibers (filled in black) used for CSA measurement. To the right, a histogram displays the frequency distribution (%) of fiber CSA for all annotated fibers. All magnifications were acquired at 100 **×** magnification; scale bar = 100 μm. Fiber CSA was measured for 100 fibers per group.


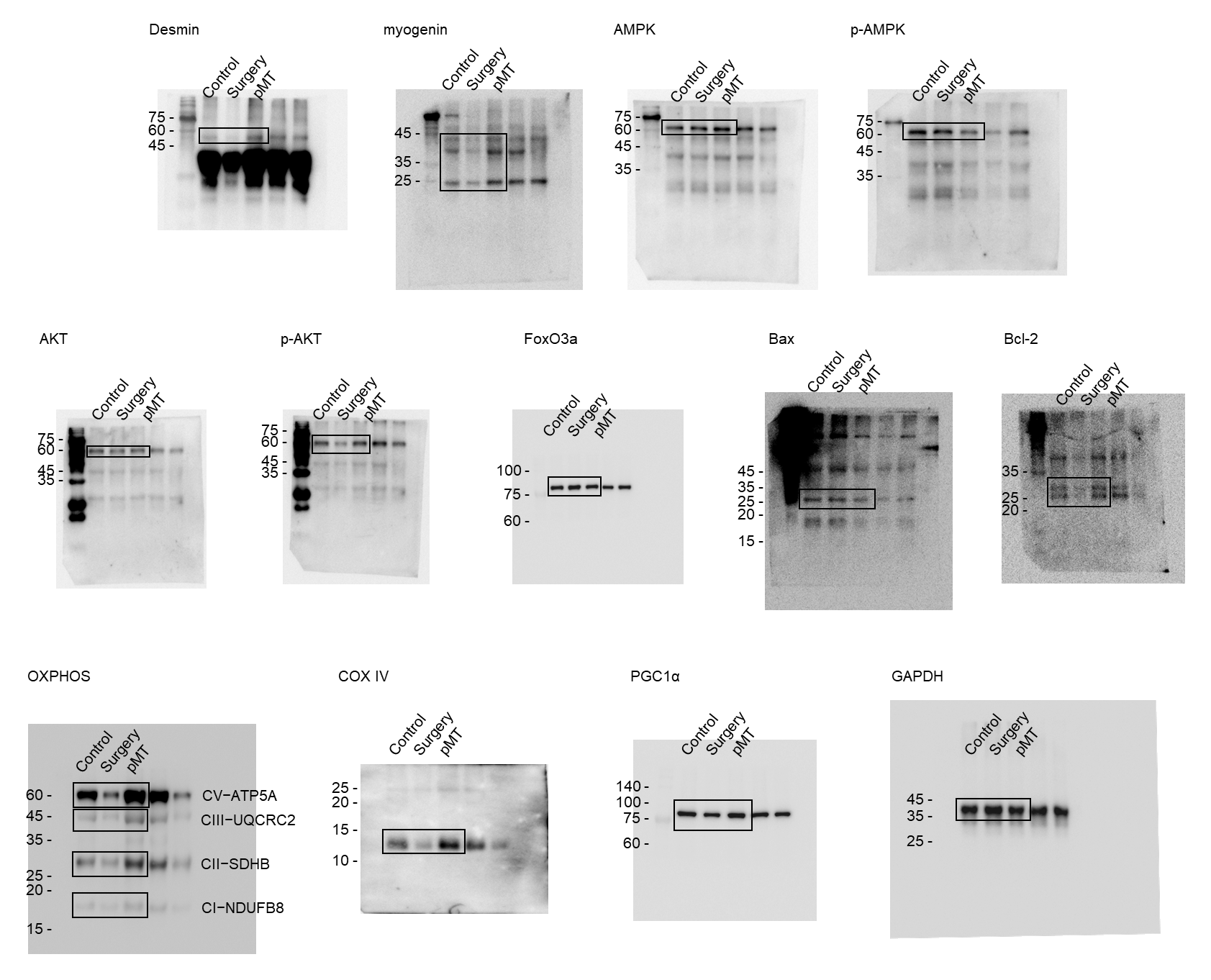


**Supplemental Figure 7.** Uncropped images from Western blots. Images are shown that correspond to Figure 5A.
